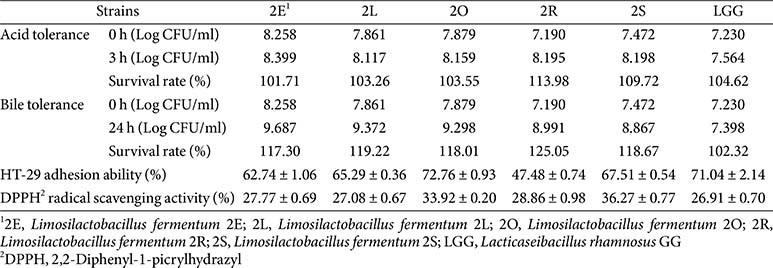# *Limosilactobacillus fermentum* 2L Ameliorates Chronic Stress-Induced Neuroinflammation through Gut-Brain Axis Modulation in Mice

**DOI:** 10.4014/jmb.2509.09035

**Published:** 2025-11-26

**Authors:** Jae Yeon Joung, Sejin Cheon, Jae Gwang Song, Chaeeun Han, Jeong Seok So, Jong Kook Moon, Hyung Wook Kim, Sae Hun Kim

**Affiliations:** 1 College of Life Sciences and Biotechnology, Korea University, Seoul 02841, Republic of Korea; 2 Institute of Life Sciences and Natural Resources, Korea University, Seoul 02841, Republic of Korea; 3 Department of Integrative Bioscience and Biotechnology, Sejong University, Seoul 05006, Republic of Korea; 4 BTSynergy Co., Ltd., Cheongju 28116, Republic of Korea

**Keywords:** *Limosilactobacillus fermentum* 2L, probiotics, gut-brain axis, chronic stress, neuroinflammation, microbiota modulation

## Abstract

Chronic stress contributes to neuroinflammation and psychiatric disorders through gut-brain axis dysregulation. This study investigated the therapeutic potential of *Limosilactobacillus fermentum* 2L in ameliorating stress-induced neuroinflammation using an unpredictable chronic mild stress (UCMS) model. Five *L. fermentum* strains were screened for probiotic properties, with strain 2L selected based on superior acid and bile tolerance, cellular adhesion, and antioxidant activity. C57BL/6J mice underwent 7-week UCMS with concurrent 10-weeks of 2L treatment (10^9^ CFU/day). Behavioral assessments, histological analysis, qRT-PCR, Western blotting, and gut microbiome analysis were performed. Strain 2L demonstrated superior gastrointestinal survival and anti-inflammatory properties in LPS-challenged HT-29 cells. In UCMS mice, 2L treatment significantly ameliorated anxiety- and depression-like behaviors, preserved hippocampal neuronal morphology, and normalized hypothalamic-pituitary-adrenal (HPA) axis dysfunction by reducing elevated corticosterone levels (155.9 ± 17.2 to 121.3 ± 3.1 ng/ml, *p* < 0.001). Molecular analysis revealed restored hippocampal BDNF expression, normalized serotonin receptors (HTR1A, 5HT7R), and attenuated stress-activated MAPK pathways (ERK1/2, JNK1/2). Treatment restored intestinal barrier integrity through tight junction protein upregulation and reduced pro-inflammatory cytokine expression. Microbiome analysis showed successful *Limosilactobacillus* colonization with restoration of beneficial bacteria (*Faecalibaculum*, *Akkermansia*) and normalization of stress-elevated *Prevotella*. *L. fermentum* 2L provides multifaceted neuroprotection through gut-brain axis modulation, involving microbiota restoration, intestinal barrier strengthening, HPA axis normalization, and enhanced neuroplasticity. These findings support the therapeutic potential of targeted probiotic interventions for stress-related neuropsychiatric disorders.

## Introduction

Chronic stress represents a major public health challenge, contributing to the pathogenesis of numerous psychiatric and neurological disorders, including major depressive disorder, anxiety disorders, and neurodegenerative diseases [1]. The prevalence of stress-related mental health conditions has escalated markedly in recent years. According to the World Health Organization, approximately 332 million people worldwide suffer from depression, and estimates for anxiety disorders have reached over 359 million cases globally [2]. Current therapeutic approaches, primarily based on pharmacological interventions targeting neurotransmitter systems, often provide incomplete symptom relief and are associated with significant side effects, highlighting the critical need for novel therapeutic strategies [3, 4].

The gut-brain axis has emerged as a pivotal bidirectional communication pathway linking intestinal function with central nervous system activity, offering novel insights into the pathophysiology and treatment of stress-related disorders [5, 6]. This complex network involves neural, hormonal, and immunological signaling mechanisms that facilitate bidirectional communication between the gastrointestinal tract and brain [7]. Accumulating evidence suggests that gut microbiota composition and metabolic activity play crucial roles in modulating mood, behavior, and stress responses, establishing the microbiota-gut-brain axis as a promising therapeutic target [5, 8].

Chronic stress profoundly disrupts gut microbiota homeostasis and compromises intestinal barrier integrity, establishing a pathological cycle that perpetuates systemic inflammation and behavioral dysfunction [9]. Stress-induced alterations in the hypothalamic-pituitary-adrenal (HPA) axis lead to elevated glucocorticoid levels, which can compromise intestinal epithelial integrity and promote bacterial translocation [10]. This increased intestinal permeability facilitates the systemic entry of pro-inflammatory bacterial lipopolysaccharides and metabolites, triggering inflammatory cascades that ultimately impair neuronal function and behavior [11].

The hippocampus, critically involved in learning, memory consolidation, and emotional regulation, exhibits particular vulnerability to chronic stress-induced pathology [12]. Prolonged stress exposure induces hippocampal neuronal atrophy, reduces neuroplasticity markers including brain-derived neurotrophic factor (BDNF), and dysregulates neurotransmitter systems, particularly serotonergic signaling pathways [13]. These molecular and structural changes contribute to the cognitive and affective symptoms characteristic of stress-related psychiatric disorders.

Probiotics have gained considerable attention as potential therapeutic agents for stress-related disorders [14, 15]. Specific probiotic strains, termed psychobiotics, demonstrate the capacity to modulate brain function and behavior through various mechanisms, including neurotransmitter production, immune system modulation, and HPA axis regulation [16]. Clinical studies have shown that specific probiotic interventions can improve mood, reduce anxiety, and enhance stress resilience in both healthy individuals and patients with psychiatric disorders [17, 18]. *Limosilactobacillus fermentum* represents a promising probiotic species with documented anti-inflammatory, antioxidant, and immunomodulatory properties [19]. This species has been isolated from various fermented foods and human gastrointestinal tract, demonstrating good colonization potential and safety profile [20]. Previous studies have reported beneficial effects of *L. fermentum* strains in various disease models, including inflammatory bowel disease, metabolic disorders, and immune dysfunction, suggesting broad therapeutic potential [19, 21]. However, the specific effects of *L. fermentum* on stress-related neuroinflammation and the underlying mechanisms of gut-brain axis modulation remain poorly characterized. The considerable inter-strain variability in probiotic efficacy necessitates comprehensive strain-specific evaluation to identify optimal therapeutic candidates [22]. Furthermore, the complex interactions between microbiota modulation, intestinal barrier function, systemic inflammation, and neuroplasticity require comprehensive mechanistic investigation to understand how probiotics exert their psychoactive effects.

Therefore, this study aimed to systematically evaluate the therapeutic potential of *L. fermentum* strains in ameliorating chronic stress-induced neuroinflammation and behavioral dysfunction. We hypothesized that specific *L. fermentum* strains would provide neuroprotective effects through comprehensive gut-brain axis modulation, involving restoration of gut microbiota homeostasis, strengthening of intestinal barrier function, normalization of HPA axis activity, and enhancement of hippocampal neuroplasticity. Through systematic strain screening, comprehensive behavioral and molecular analyses, and detailed microbiome profiling, this research provides mechanistic insights into probiotic-mediated neuroprotection and establishes the foundation for clinical translation of targeted psychobiotic interventions.

## Materials and Methods

### Preparation and Selection of Probiotics Strain

The bacterial strains used in this study were prepared from the Food Microbiology Laboratory, Department of Food Bioscience and Technology, Korea University (Republic of Korea). *L. fermentum* strains were screened using the experiments below. The strains were cultured for 18 h at 37°C de Man, Rogosa and Sharpe (MRS) broth (BD Co., USA) before every use.

### Determination of Acid and Bile Tolerance Activities

The acid and bile tolerance of strains was evaluated as described previously by Oh *et al*. [23] with slight modification. In brief, cultured *Lactobacillus* strains were transferred into MRS broth (pH 2.5) by adding 1,000 units/ml of pepsin (Sigma Aldrich, USA), and MRS broth (pH 6.5) with 0.3% oxgall (Sigma Aldrich) for testing the acid and bile tolerance, respectively. The tolerance activities were determined by comparing the colony-forming units (CFU) of the strains using the pour-plating method on MRS agar immediately after inoculation with the CFU of those after 3 h with MRS (pH 2.5) and for 48 h with MRS (pH 6.5).

### Determination of Adhesion Ability

For the assessment of adhesion ability to the intestinal epithelium, the human colon cancer cell line HT-29 was used and the assay was performed using the method described by Oh *et al*. [23]. The adhesion ability was assessed by count the CFU using the pour-plating method on MRS agar.

### Determination of Antioxidant Activity

To assess the antioxidant capability of *Lactobacillus* strains, the 2,2-diphenyl-1-picrylhydrazyl (DPPH) assay was performed [23]. Mixture of 800μl of cultured strains and 1 ml of 0.2 mM DPPH was allowed to incubate in the dark for 30 min. Following incubation, the absorbance of strain was measured at 517 nm with Microplate Reader (Versa Max Microplate Reader, Molecular Device, USA).

### Cell-Based *In Vitro* Assay

**Cell culture and treatment.** HT-29 human epithelial cells were obtained from the Korean Cell Line Bank (KCLB, Republic of Korea). The HT-29 cells were grown on plastic cell culture dishes in a cell 37°C, 5% CO_2_ incubator with RPMI 1640 medium (Gibco, USA) supplemented with 10% heat-inactivated FBS and 1%penicillin/streptomycin. The growth medium was changed every 48 h.

**Cytotoxicity CCK-8 assay.** Cell counting kit-8 (CCK-8) assay was carried out using CyQUANT LDH Cytotoxicity Assay Kit (APExBIO Technology LLC, USA), as previously described [24]. In brief, HT-29 cells were seeded in a 96-well plate at a concentration of 1×10^4^ cells/well, and treated with different concentrations of probiotic strains and incubated at 37°C with 5% CO_2_ for 24 h. Control group was also prepared for comparison. After incubation, 10 μl of the CCK-8 solution was added to each well of the plate. The wells were then incubated in the CO_2_ incubator for an additional 1 h. The absorbance was measured at 450 nm using a BioTek epoch (Agilent Technologies, USA) to calculate cytotoxicity.

**Anti-inflammatory activity.** HT-29 cells were cultured on a 96-well plate and incubated at 37°C with 5% CO_2_. The culture medium used was Gibco RPMI 1640, supplemented with 10% heat-inactivated FBS and 1% penicillin/streptomycin. After incubation for 24 h, the HT-29 cells were exposed to *Lactobacillus* strains and incubated for an additional 24 h. Subsequently, each well was treated with 100 μl of 5 μg/ml lipopolysaccharide (LPS) and incubated for another 24 h. Finally, the HT-29 cells were harvested, and the expression levels of inflammatory cytokines and tight junction proteins were analyzed using quantitative reverse transcription PCR (qRT-PCR).

### Quantitative Reverse Transcription PCR (qRT-PCR)

The mRNA extraction and qRT-PCR were carried out as the method described by Joung *et al*. [25]. Total mRNA was extracted from cells and tissues, using TRIzol reagent (Invitrogen, USA) following the manufacturer's instructions and cDNA was synthesized by the reverse transcription kit (Thermo Fisher Scientific, USA). Subsequently, qRT-PCR was performed with the Biorad CFX96 Real-Time PCR Detection System (Bio-Rad, USA). Targeted genes were quantified using MG2 x qPCR MasterMix (SYBR green) (MGmed, Republic of Korea), and the qRT-PCR cycling conditions were initial denaturation cycle at 95°C for 10 min followed by 40 cycles of amplification at 95°C for 15 sec, annealing at 55-65°C for 30 sec, and extension at 70°C for 5 sec. The mRNA expression levels of each targeted genes were analyzed and normalized to the internal standard gene GAPDH using Bio-Rad CFX Maestro (Bio-Rad). The primer sequences used in this study are listed in Table S1. The relative mRNA expression was calculated using the comparative threshold cycle method, normalized to the GAPDH expression level, and quantified using the 2-ΔΔCt method [26].

### Animal Experiment

A total of 36 C57BL/6 male mice (Samtako Bio Korea Co. Ltd., Republic of Korea) were procured at 5 weeks of age. After acclimation for 1 week, they were housed in groups of four mice per cage and randomly assigned to three different groups (*n* = 12 in each group): nonstress group with PBS (CON), unpredictable chronic mild stress group with PBS (UCMS), and UCMS with *L. fermentum* 2L (1 × 10^10^ CFU/kg/day) group (2L). The sample size (*n* = 12 per group) was determined based on previous studies in the gut–brain axis and neuroinflammation fields that successfully detected significant behavioral and inflammatory effects with comparable or smaller group sizes (*n* = 8–10 per group) [14, 27]. The mice were kept in filter top-covered polycarbonate cages (22 cm wide × 28 cm long × 13 cm tall) with a stainless-steel lid under standard conditions (12-h light-dark cycle, 23 ± 2°C, 50 ± 10% relative humidity). After 2 weeks of sample pretreatment, the UCMS and 2L groups of mice were subjected to various stressors in a random order, including changes in sleep cycles, wet bedding, tilted cages, illumination, water deprivation, restraint, and cold-water baths over the course of 6 weeks. The CON group remained undisturbed, except for routine housekeeping procedures. At the end of week 8, the mice were euthanized by anesthetize them with isoflurane for approximately 120 sec, followed by cardiac puncture to collect blood. Fecal samples from mice were freshly collected on week 0 and week 8. Ileum, colon, and brain tissues were dissected and stored at −20°C until physiological analyses were performed. All mouse experiments were approved by the Institutional Animal Care and Use Committee of Sejong University (SJ-20230110-01) and were conducted in accordance with the guidelines for the Care and Use of Laboratory Animals.

### Behavioral Test

**Open-field test.** The open-field apparatus consisted of a square arena (40 cm × 40 cm) with 30-cm-high walls. Mice were placed in one corner and allowed to explore freely for 20 min. Locomotor activity, exploratory behavior, and anxiety-like behavior were recorded and analyzed using ANY-maze software (Stoelting, USA). Between trials, the apparatus was cleaned with 70% ethanol and dried completely [28].

**Elevated plus-maze test.** The elevated plus-maze consisted of two open arms (5 cm wide × 30 cm long) and two closed arms (5 cm wide × 30 cm long × 15 cm high walls) arranged in a cross configuration, elevated 40 cm above the floor. Each mouse was placed in the center facing a closed arm and allowed to explore for 5 min. Time spent in open arms, closed arms, and number of arm entries were recorded using ANY-maze software. Anxiety index was calculated as follows:


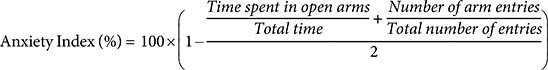


Increased time in open arms indicates reduced anxiety-like behavior, while preference for closed arms suggests heightened anxiety [29].

**Tail suspension test.** Mice were suspended by their tails using adhesive tape positioned approximately 1 cm from the tail tip, at a height of 50 cm above the floor. The test duration was 6 min, and immobility time was recorded during the final 4 min. Immobility was defined as the absence of escape-oriented movements. Increased immobility time indicates depression-like behavior [30].

**Forced swim test.** Mice were placed individually in transparent cylindrical containers (20 cm diameter × 25 cm height) filled with water (24 ± 1°C) to a depth of 15 cm for 6 min. Immobility time was measured during last 4 min of the 6-min session. Immobility was defined as floating motionless or making only minimal movements necessary to keep the head above water. Increased immobility time reflects depression-like behavior [31].

### Histological Analysis

Upon completing the study, histological analysis was performed on the colon and the brain samples obtained from each animal. Colon and brain samples from each group were fixed in 10% (v/v) formaldehyde, then dehydrated in ethanol, and subsequently embedded in paraffin using standard techniques. The samples were sectioned into 4-5 μm thick slices and stained with hematoxylin and eosin (H&E). Digital photomicrographs were taken from representative areas using a digital camera and observed using Image J (National Institutes of Health, USA).

### Serum Analysis

Blood samples were collected in BD Vacutainer SST II Advance tubes (Becton Dickinson, USA) after cardiac puncture and were stored at −20°C for 30 min. Serum corticosterone and serotonin levels were measured using enzyme- linked immunosorbent assay (ELISA) kits (Abcam, USA), according to the manufacturer’s instructions. The quantification of β-endorphin was performed using a Luminex assay with the MILLIPLEX Rat/Mouse Neuropeptide Magnetic Bead Panel (Merck, Germany) according to the manufacturer’s instructions. All measurements were performed in triplicate.

### Western Blot Analysis

Protein was extracted from dissected hippocampus tissues and the western blotting was carried out as described previously [14]. In brief, the total protein (1 mg/ml) was separated by sodium dodecyl sulfate-polyacrylamide gel electrophoresis, and then the resolved proteins were transferred to a polyvinylidene difluoride membrane. The membrane was incubated with a targeted primary antibody, followed by the appropriate horseradish peroxidase-conjugated secondary antibody. Protein bands were visualized using a ImageQuant LAS 4000 mini (GE-Healthcare). Protein expression was quantified using ImageJ software (National Institutes of Health) and normalized against that of β-actin. The antibodies used are listed in Table S2.

### Fecal Microbiome Analysis

Fecal genomic DNA isolation and sequencing, along with taxonomic analysis, were conducted following the method described by Eor *et al*. [32] with slight modifications. Genomic DNA was extracted from fecal samples using the QIAamp DNA Stool Mini Kit (QIAGEN, Germany) according to the manufacturer’s instructions. Double-stranded DNA concentration was quantified using Quant-iT PicoGreen (Invitrogen). For 16S rRNA gene sequencing, PCR amplification targeting the V3-V4 hypervariable regions was performed using the following universal primers: V3-F (5'-TCG TCG GCA GCG TCA GAT GTG TAT AAG AGA CAG CCT ACG GGN GGC WGC AG-3') and V4-R (5'-GTC TCG TGG GCT CGG AGA TGT GTA TAA GAG ACA GGA CTA CHV GGG TAT CTA ATC C-3'). Metagenomic libraries were prepared using the Nextera XT Kit (Illumina, USA) following the manufacturer’s protocol.

Sequencing was performed on an Illumina MiSeq platform (MiSeq R2500) using 2×300 bp paired-end chemistry on multiplexed pooled samples. All sequencing procedures were conducted by Macrogen Inc. (Republic of Korea). Sequencing yielded an average of approximately 130,000 raw reads per sample (range: 99,206-155,846), with all samples achieving >90% of bases with a quality score of Q20 or higher, indicating high sequencing quality.

Raw sequencing data were processed and analyzed using Quantitative Insights into Microbial Ecology 2 (QIIME2, 2024.10) pipeline [33]. Paired-end demultiplexed sequences were imported using the ‘qiime tools import’ command. Quality filtering, denoising, and chimera removal were performed using the DADA2 plugin (‘q2-dada2 denoise-paired’ command) with default parameters. After quality filtering and denoising, 24 samples were retained, with an average of 17,613 high-quality sequences per sample (range: 13,367-22,673; median 17,660). Amplicon sequence variants (ASVs) were generated through the DADA2 pipeline. Taxonomic classification was conducted using a pre-trained Naïve Bayes classifier trained on the SILVA v132 reference database (99% OTUs) targeting the V3-V4 region of the 16S rRNA gene (‘qiime feature-classifier classify-sklearn’ command). To minimize bias caused by uneven sequencing depth, all samples were rarefied to 13,000 sequences per sample, corresponding to the minimum observed sequencing depth, for subsequent alpha and beta diversity analyses. Taxonomic composition was visualized using QIIME2 commands such as ‘qiime metadata tabulate’ and ‘qiime taxa barplot’ with the SILVA v132 classifier.

### Statistical Analysis

All experiments were performed in triplicate. The data are expressed as the means ± standard error of the mean (SEM). Statistical analysis was carried out using IBM SPSS statistics software version 25.0 (IBM, USA). One-way analysis of variance (ANOVA) with Duncan’s test or student’s *t*-test was used to analyze the statistical difference between the mean values of samples. Statistical significance was defined as *p* < 0.05.

## Results

### Selection and Characterization of *L. fermentum* 2L as Probiotic Strain

Five *L. fermentum* strains (2E, 2L, 2O, 2R, and 2S) were systemically evaluated for key probiotic properties, including acid and bile tolerance, cellular adhesion ability, and antioxidant activity (Table 1). All tested strains demonstrated excellent acid tolerance, maintain viability above 101% after 3 h of exposure to pH 2.5 conditions, which was comparable to the reference strain *L. rhamnosus* GG (LGG; 104.62%). Under bile stress conditions, the *L. fermentum* strains showed superior bile tolerance compared to LGG. Strains 2R, 2L, 2O, 2E, and 2S demonstrated survival rates of 125.05%, 119.22%, 118.01%, 117.30%, and 118.67%, respectively, substantially exceeding that of LGG (102.32%), indicating robust gastrointestinal survival capabilities.

Adhesion to HT-29 human intestinal epithelial cells varied among strains, ranging from 47.48 ± 0.74% (2R) to 72.76 ± 0.93% (2O). Strains 2E, 2L, 2O, and 2S exhibited adhesion rates of 62.74 ± 1.06%, 65.29 ± 0.36%, 72.76 ± 0.93%, and 67.51 ± 0.54%, respectively, which were slightly lower than LGG (71.04 ± 2.14%). Strain 2R showed significantly reduced adhesion capacity, making it less suitable for further development. All *L. fermentum* strains demonstrated superior DPPH radical scavenging activity compared to LGG (26.91 ± 0.70%), with antioxidant activities ranging from 27.08 ± 0.67% (2L) to 36.27 ± 0.77% (2S). Based on comprehensive evaluation of probiotic characteristics, strain 2L was selected for subsequence investigations due to its balanced performance across all tested parameters.

### Anti-Inflammatory Effects of *L. fermentum* 2L in LPS-Challenged HT-29 Cells

**Fig. 1:**
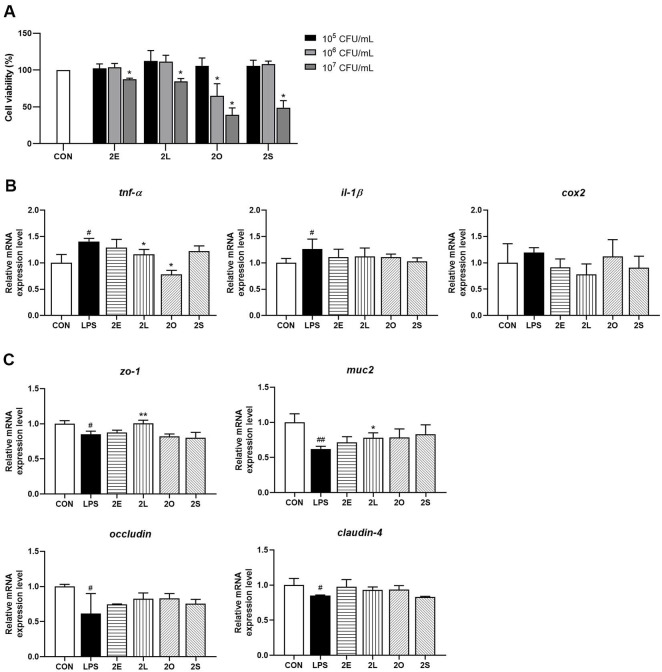
*In vitro* screening and anti-inflammatory effects of *L. fermentum* strains in HT-29 cells. (**A**) Cell viability assessment of HT-29 intestinal epithelial cells following treatment with five *L. fermentum* strains. (**B**) Quantitative analysis of pro-inflammatory cytokine gene expression in HT-29 cells. (**C**) Expression analysis of intestinal barrier integrity genes in HT- 29 cells. Data are presented as mean ± SEM from three independent experiments. Statistical significance was determined by one-way ANOVA followed by Tukey's multiple comparison test. *p < 0.05, **p < 0.005 compared to LPS-treated group. CON, untreated control; LPS, lipopolysaccharide-treated positive control; 2E, 2L, 2O, 2S, *L. fermentum* strain.

Cytotoxicity assessment using the CCK-8 assay revealed that treatment with 2E and 2L at concentrations ranging from 10^5^ to 10^7^ CFU/ml for 24 h showed no significant cytotoxic effects on HT-29 cells, with cell viability maintained above 80% at all tested concentrations (Fig. 1A).

The anti-inflammatory properties of the four strains were investigated using an LPS-induced inflammatory model in HT-29 cells. LPS stimulation significantly upregulated pro-inflammatory cytokine expression, as evidenced by increased *tnf-α* mRNA levels compared to controls. Particularly, pre-treatment with 2L effectively attenuated this inflammatory response, significantly reducing *tnf-α* expression compared to the LPS-only group (*p* < 0.05) (Fig. 1B). In contrast, *il-1β* and *cox2* expression levels showed no significant changes following 2L treatment, suggesting a selective anti-inflammatory mechanism.

To evaluate the impact of the strains on intestinal barrier function, the expression of key tight junction and mucin proteins was analyzed. LPS challenge significantly downregulated expression of tight junction proteins *zo-1* and *ocldn*, as well as the mucin gene *muc2* (Fig. 1C). Treatment with 2L substantially restored expression of these barrier-associated genes, significantly increasing *zo-1* mRNA levels (*p* < 0.005) and *muc2* expression (*p* < 0.05) compared to the LPS-treated group. However, *ocldn* and *cldn-4* expression remained unchanged across all treatment groups. These results demonstrate that 2L exhibits protective effects against LPS-induced inflammation while maintaining intestinal epithelial barrier integrity.

### Behavioral and Neuroprotective Effects of *L. fermentum* 2L in UCMS-Induced Stress Mice

**Fig. 2:**
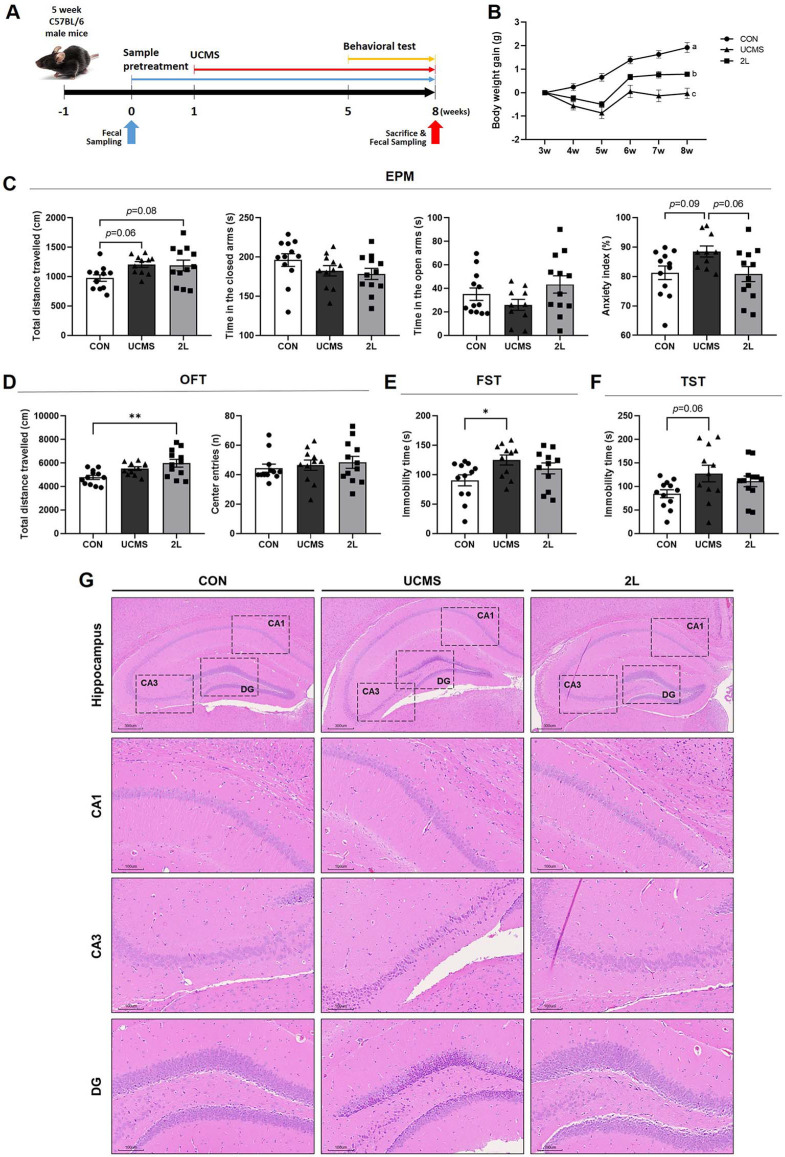
*L. fermentum* 2L treatment ameliorates chronic stress-induced behavioral deficits and preserves hippocampal morphology. (**A**) Experimental timeline for the unpredictable chronic mild stress (UCMS) model. (**B**) Body weight changes throughout the experimental period. (**C**) Elevated plus maze (EPM) test. (**D**) Open field test (OFT). (**E**) Forced swim test (FST). (**F**) Tail suspension test (TST). (**G**) Representative hematoxylin and eosin (H&E) stained hippocampal sections showing morphological changes in different brain regions. Images display hippocampus (scale bar: 500 μm), CA1 pyramidal cell layer, CA3 pyramidal cell layer, and dentate gyrus (DG) granule cell layer (scale bar: 100 μm). Data are presented as mean ± SEM (*n* = 10). Statistical significance was determined by one-way ANOVA followed by Tukey's multiple comparison test. *p < 0.05, **p < 0.005, ***p < 0.001 compared to control group; #*p* < 0.05, ##*p* < 0.005 compared to UCMS group. CON, control group; UCMS, unpredictable chronic mild stress group; 2L, UCMS + *L. fermentum* 2L treatment group.

To evaluate the therapeutic effects of 2L on stress-related disorders, mice were subjected UCMS for 7 weeks with concurrent probiotic treatment (Fig. 2A). Body weight monitoring throughout the experimental period showed significant decrease in the UCMS group compared to controls, which was restored in the 2L-treated group (Fig. 2B).

Anxiety and depression-like behaviors were evaluated using EPM, OFT, FST, and TST. In the EPM, UCMS exposure reduced exploratory behavior, as evidenced by an increased anxiety index (trend toward significance, *p* = 0.09) compared to controls, which was ameliorated by 2L pretreatment (trend toward improvement, *p* = 0.06)(Fig. 2C). The OFT revealed that 2L treatment significantly increased total distance traveled compared to the UCMS group, indicating enhanced locomotor activity (*p* < 0.005) (Fig. 2D). In the FST, UCMS significantly increased immobility time compared to controls (*p* < 0.05), indicating enhanced depression-like behavior (Fig. 2E). Treatment with 2L showed a trend toward reduced immobility time compared to the UCMS group. Similarly, in the TST, UCMS increased immobility time (trend, *p* = 0.06), while 2L treatment tended to decrease this response (Fig. 2F).

Histological analysis of hippocampal sections using H&E staining evaluated the neuroprotective effects of 2L treatment (Fig. 2G). Control mice displayed normal hippocampal morphology with uniformly sized cell bodies and regular distribution patterns in CA1, CA3, and dentate gyrus (DG) regions. UCMS exposure induced marked morphological alterations, including cellular shrinkage, irregular cell shapes, and reduced cell density across all examined regions. The CA3 region showed particularly severe structural disruption with apparent neuronal loss and disorganized cellular arrangement. Treatment with 2L subsequently attenuated UCMS-induced hippocampal pathology, with neuronal morphology showing improved cell size and more regular distribution patterns compared to the UCMS group.

**Fig. 3:**
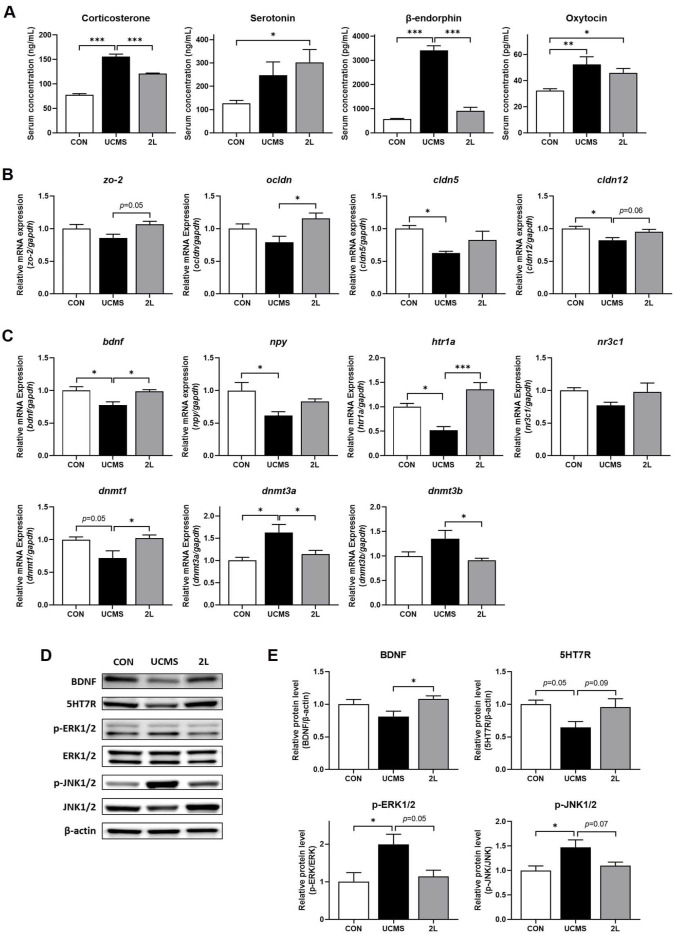
*L. fermentum* 2L normalizes stress-induced alterations in neuroendocrine signaling and hippocampal gene expression. (**A**) Serum levels of stress-related hormones and neuropeptides measured by ELISA. (**B**) Hippocampal expression of tight junction and barrier function genes assessed by qRT-PCR. (**C**) Hippocampal expression of neuroplasticity and neurotransmitter-related genes. (**D**) Representative Western blot images showing protein expression of key signaling molecules in hippocampal tissue. (**E**) Quantitative analysis of Western blot results. Protein levels were normalized to β-actin and expressed as fold change relative to control group. Data are presented as mean ± SEM (*n* = 10). Statistical significance was determined by one-way ANOVA followed by Tukey's multiple comparison test. *p < 0.05, **p < 0.005, ***p < 0.001 compared to control group; #*p* < 0.05, ##*p* < 0.005 compared to UCMS group. CON, control group; UCMS, unpredictable chronic mild stress group; 2L, UCMS + *L. fermentum* 2L treatment group.

*L. fermentum* 2L Regulates Hippocampal Neuroplasticity and Stress Response in UCMS-Induced Stress Mice To assess the impact of 2L on the HPA axis, serum levels of stress-related hormones and neurotransmitters were measured (Fig. 3A). UCMS exposure significantly elevated serum corticosterone levels (155.9 ± 17.2 ng/ml) compared to controls (78.0 ± 7.9 ng/ml, *p* < 0.001), indicating HPA axis hyperactivation. This elevation was accompanied by increased β-endorphin (3412.0 ± 546.9 pg/mL vs. 577.7 ± 59.4 pg/ml in controls, *p* < 0.001) and oxytocin levels (52.5 ± 19.3 pg/ml vs. 32.3 ± 5.2 pg/ml in controls, *p* < 0.005), suggesting compensatory mechanisms for stress response.

Treatment with 2L significantly normalized these stress-induced hormonal alterations. Corticosterone levels were reduced to 121.3 ± 3.1 ng/ml (*p* < 0.001 vs. UCMS), while β-endorphin was decreased to 913.7 ± 421.3 pg/ml (*p* < 0.001 vs. UCMS). The UCMS-induced elevation of oxytocin was also attenuated by 2L pretreatment. Notably, serotonin concentrations were significantly increased in the 2L group (302.8 ± 191.2 ng/ml) compared to controls (127.5 ± 41.4 ng/ml, *p* < 0.05), suggesting enhanced serotonergic neurotransmission.

In hippocampal tissues, 2L treatment effectively preserved blood-brain barrier integrity by upregulating tight junction proteins (Fig. 3B). Expression of *zo-2* showed a trend toward increase (*p* =0.05), while *ocldn* (*p* < 0.05) and *cldn12* (*p* = 0.06) were upregulated compared to the UCMS group, indicating comprehensive restoration of barrier function.

Analysis of neurotrophic and neuroplasticity markers revealed significant alterations following UCMS exposure (Fig. 3C). UCMS significantly reduced mRNA expression of brain-derived neurotrophic factor (*bdnf*, *p* < 0.05) and neuropeptide Y (*npy*, *p* < 0.05) in the hippocampus, indicating impaired neuroplasticity and stress resilience. The serotonin receptor *htr1a* expression was significantly decreased upon the induction of stress (*p* < 0.05), while 2L treatment markedly restored its expression (*p* < 0.001 vs. UCMS). Similarly, the glucocorticoid receptor *nr3c1* showed reduced expression following stress induction, which was restored by 2L treatment. UCMS exposure dysregulated the expression of DNA methyltransferases (DNMTs), key enzymes involved in epigenetic regulation. Specifically, *dnmt1* (*p* =0.05), *dnmt3a* (*p* < 0.05), and *dnmt3b* (trend) showed altered expression patterns following UCMS. Treatment with 2L significantly normalized these epigenetic regulators compared to the UCMS group (*p* < 0.05).

Western blot analysis of hippocampal tissues confirmed the qRT-PCR findings at the protein level (Fig. 3D and 3E). BDNF protein levels, which showed a trend toward reduction in UCMS mice, were significantly restored following 2L treatment (*p* < 0.05). Similarly, serotonin receptor 7 (5HT7R) protein levels, decreased by UCMS (*p* = 0.05), showed improvement in the 2L group (*p* = 0.09). Analysis of stress-activated protein kinases revealed that UCMS significantly increased phosphorylation of extracellular signal-regulated kinases 1/2 (p-ERK1/2, *p* < 0.05) and c-Jun N-terminal kinases 1/2 (p-JNK1/2, *p* < 0.05) compared to controls, indicating chronic stress signaling activation. Treatment with 2L attenuated these stress-activated pathways, reducing p-ERK1/2 (*p* = 0.05) and p-JNK1/2 (*p* = 0.07) compared to UCMS mice.

These comprehensive neurobiological analyses demonstrate that 2L exerts multifaceted neuroprotective effects through modulation of the HPA axis, enhancement of neurotrophic signaling, preservation of blood-brain barrier integrity, and attenuation of stress-activated kinase pathways.

### Modulation of Intestinal Inflammatory and Barrier Function by *L. fermentum* 2L in UCMS-Induced Stress Mice

**Fig. 4:**
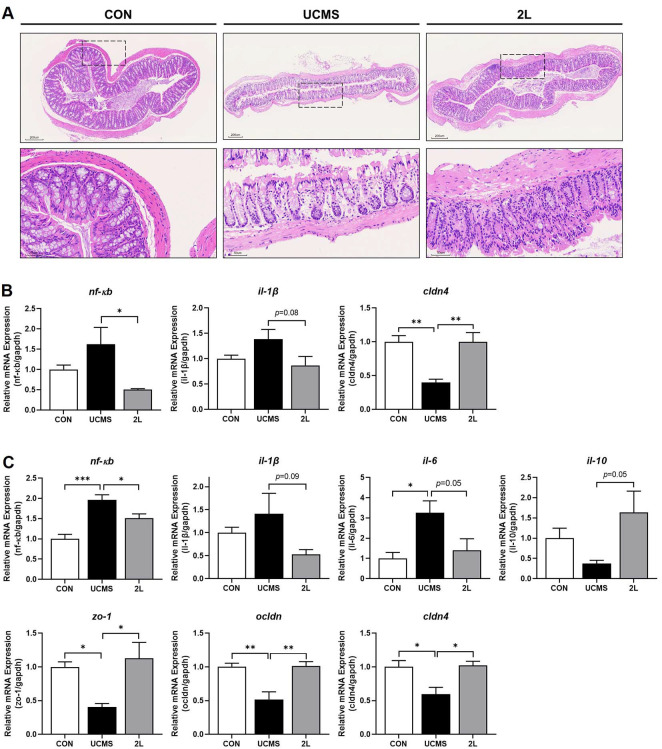
*L. fermentum* 2L restores intestinal barrier integrity and reduces gut inflammation in UCMS mice. (**A**) Representative hematoxylin and eosin (H&E) stained sections of duodenal tissue showing morphological changes in intestinal architecture. Upper panels show low magnification overview of duodenal structure (scale bar: 200 μm), while lower panels display high magnification images of intestinal villi and crypts (scale bar: 50 μm). (**B**) Colonic expression of pro-inflammatory cytokines and barrier function genes assessed by qRT-PCR. (**C**) Ileal expression of inflammatory and barrier integrity markers assessed by qRT-PCR. Data are presented as mean ± SEM (*n* = 10). Statistical significance was determined by one-way ANOVA followed by Tukey's multiple comparison test. *p < 0.05, **p < 0.005, ***p < 0.001 compared to control group; #*p* < 0.05, ##*p* < 0.005 compared to UCMS group. CON, control group; UCMS, unpredictable chronic mild stress group; 2L, UCMS + *L. fermentum* 2L treatment group.

Histological analysis of colonic tissues revealed significant UCMS-induced structural alterations and their amelioration following 2L treatment (Fig. 4A). H&E staining of colonic sections from control mice showed well-formed villous architecture with minimal inflammatory cell infiltration and intact epithelial integrity. In contrast, UCMS exposure induced severe intestinal pathology characterized by villous atrophy, structural collapse, and pronounced inflammatory cell infiltration throughout the mucosa and submucosa. Treatment with 2L markedly preserved intestinal architecture, maintaining well-organized villous structures and significantly reducing inflammatory cell infiltration compared to the UCMS group.

qRT-PCR analysis of colonic tissues revealed profound alterations inflammatory mediators and tight junction proteins following UCMS and 2L treatment (Fig. 4B). UCMS exposure upregulated pro-inflammatory signaling, with increased *nf-κb* and *il-1β* expression compared to controls. Concomitantly, the tight junction protein *cldn4* was significantly downregulated (*p* < 0.005), indicating compromised barrier function. Treatment with 2L effectively counteracted these inflammatory changes. Expression of *nf-κb* was significantly reduced (*p* < 0.05), while *il-1β* showed a trend toward normalization (*p* = 0.08) compared to the UCMS group. Most notably, *cldn4* expression was significantly restored (*p* < 0.005), suggesting improved epithelial barrier integrity.

Similar inflammatory patterns were observed in ileal tissues, with more pronounced alterations compared to colonic samples (Fig. 4C). UCMS significantly upregulated pro-inflammatory cytokines, including *nf-κb* (*p* < 0.001) and *il-6* (*p* < 0.05), while showing trends toward increased *il-1β* and decreased anti-inflammatory il-10 expression compared to controls. Treatment with 2L effectively reversed these inflammatory changes in the ileum. Pro-inflammatory mediators were significantly reduced, with *nf-κb* (*p* < 0.05), *il-1β* (*p* = 0.09), and *il-6* (*p* = 0.05) showing decreased expression compared to UCMS mice. Additionally, the anti-inflammatory cytokine *il-10* was restored (*p* = 0.05), indicating improved inflammatory balance.

UCMS severely compromised intestinal barrier function, as evidenced by significant downregulation of key tight unction proteins in ileal tissues. Expression of *zo-1* (*p* < 0.05), *ocldn* (*p* < 0.005), and *cldn4* (*p* < 0.05) were all significantly decreased compared to controls, indicating widespread barrier dysfunction. In contrast, 2L treatment comprehensively restored tight junction protein expression. All examined tight junction components were significantly upregulated in the 2L group compared to UCMS mice: *zo-1* (*p* < 0.05) *ocldn* (*p* < 0.005), and *cldn4* (*p* < 0.05), demonstrating complete restoration of intestinal barrier integrity.

These findings demonstrate that 2L provides comprehensive intestinal protection through dual mechanisms of anti-inflammatory action and barrier function preservation, effectively counteracting UCMS-induced gastrointestinal pathology.

### Gut Microbiome Modulation by *L. fermentum* 2L in UCMS-Induced Stress Mice

**Fig. 5:**
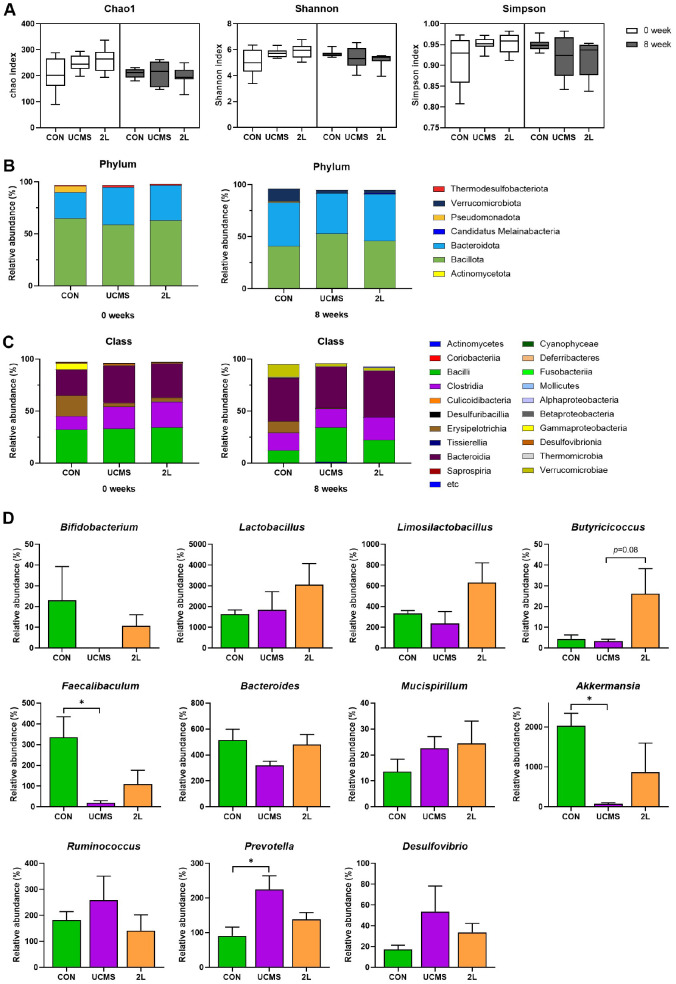
*L. fermentum* 2L treatment modulates gut microbiota composition and restores microbial diversity in UCMS mice. (**A**) Alpha diversity indices of gut microbiota assessed by 16S rRNA gene sequencing. (**B**) Phylum-level composition of gut microbiota showing relative abundance of major bacterial phyla. (**C**) Class-level taxonomic composition showing detailed bacterial community structure. (**D**) Genus-level analysis of specific bacterial taxa with therapeutic relevance. Data are presented as mean ± SEM (*n* = 10). Statistical significance was determined by one-way ANOVA followed by Tukey's multiple comparison test. *p < 0.05, **p < 0.005, ***p < 0.001 compared to control group; #*p* < 0.05, ##*p* < 0.005 compared to UCMS group. CON, control group; UCMS, unpredictable chronic mild stress group; 2L, UCMS + *L. fermentum* 2L treatment group.

Alpha diversity analysis was conducted to evaluate the effect of UCMS and 2L treatment on gut microbial community structure (Fig. 5A). The Chao1 index (species richness estimator), Shannon diversity index (accounting for both richness and evenness), and Simpson index (emphasizing dominant species) showed no significant differences between groups at both baseline (0 weeks) and post-treatment (8 weeks). These results indicate that while chronic stress and 2L treatment altered specific bacterial populations, the overall microbial richness and community evenness remained stable across all experimental conditions.

Microbiome analysis revealed distinct compositional changes across multiple taxonomic levels following chronic stress exposure. At the phylum level, baseline microbiota composition was similar across all groups, with *Bacillota* and *Bacteroidota* representing the predominant phyla (Fig. 5B). After 8 weeks of UCMS treatment, notable shifts were observed, including alterations in the *Bacillota* to *Bacteroidota* ratio and emergence of minor phyla such as *Verrucomicrobiota*.

Class-level analysis revealed more pronounced stress-induced alterations (Fig. 5C). The UCMS group exhibited a marked increase in *Clostridia* abundance with a corresponding reduction in *Bacilli* compared to controls. *Bacteroidia* relative abundance was also altered in stress-exposed animals. Treatment with 2L partially ameliorated these stress-induced changes, with *Clostridia* levels showing intermediate values between control and UCMS groups and partial restoration of *Bacilli* abundance.

Genus-level analysis identified several key bacterial taxa that were differentially affected by chronic stress and 2L treatment (Fig. 5D). Chronic stress significantly reduced beneficial bacteria including *Faecalibaculum* (*p* < 0.05) and *Akkermansia* (*p* < 0.05), while significantly increasing *Prevotella* abundance (*p* < 0.05), a genus associated with pro-inflammatory conditions. 2L supplementation demonstrated targeted therapeutic effects, resulting in substantial enrichment of *Limosilactobacillus*, confirming successful colonization of the administered probiotic strain. Additionally, 2L treatment showed a trend toward increased *Butyricicoccus* abundance (*p* = 0.08), a beneficial butyrate-producing bacterium with established anti-inflammatory properties. Importantly, probiotic supplementation partially restored stress-depleted beneficial bacteria (*Faecalibaculum* and *Akkermansia*) while normalizing the pathological elevation in *Prevotella*.

Collectively, these findings demonstrate that chronic stress induces comprehensive gut microbiota dysbiosis characterized by depletion of beneficial taxa and enrichment of potentially harmful bacteria. 2L supplementation effectively counteracts many of these stress-induced microbial perturbations, suggesting that restoration of gut microbiota homeostasis represents a key mechanism underlying its neuroprotective effects in this UCMS model.

## Discussion

This study provides comprehensive evidence that 2L ameliorates chronic stress-induced neuroinflammation through gut-brain axis modulation in mice. Our findings demonstrate that 2L exerts therapeutic effects through multiple interconnected mechanisms: restoration of gut microbiota homeostasis, strengthening of intestinal barrier function, attenuation of systemic inflammation, and normalization of neuroplasticity markers in the hippocampus.

The systematic screening approach employed for strain selection identified *L. fermentum* 2L as possessing optimal probiotic characteristics, including superior gastrointestinal survival, adequate cellular adhesion, and moderate antioxidant activity. The enhanced bile tolerance observed in all *L. fermentum* strains compared to the reference strain LGG aligns with previous reports suggesting species-specific adaptation mechanisms [34]. The moderate antioxidant activity of strain 2L is consistent with lactobacilli's capacity to produce bioactive metabolites, which may contribute to their health-promoting effects through reduction of oxidative stress [35]. The balanced profile of strain 2L across multiple functional parameters supports its suitability for therapeutic applications, as comprehensive probiotic efficacy often depends on multifactorial characteristics rather than excellence in a single property [36]. Moreover, supplementary *in vitro* experiments using corticosterone-treated SH-SY5Y cells demonstrated that the 2L strain significantly attenuated *nf-κb* expression while restoring creb and syp levels to those comparable to the untreated control (Fig. S1).

The behavioral improvements observed following 2L treatment provide compelling evidence for gut-brain axis involvement in stress-related disorders. The restoration of locomotor activity in the OFT and trends toward reduced anxiety-like behavior in the elevated plus maze align with mounting evidence that gut microbiota modulation can influence central nervous system function [5, 6]. The partial amelioration of depression-like behaviors in the FST and TST, while not reaching statistical significance, suggests that probiotic interventions may require longer treatment durations or higher doses to fully normalize affective behaviors [37, 38].

The neuroprotective effects demonstrated through hippocampal histology are particularly noteworthy, as the hippocampus is critically involved in stress response regulation and is highly vulnerable to chronic stress-induced damage [12, 39]. The preservation of neuronal morphology and density in CA1, CA3, and dentate gyrus regions following 2L treatment suggests that gut microbiota modulation can provide direct neuroprotective benefits, potentially through multiple pathways including neurotransmitter synthesis, inflammatory modulation, and neurotrophic factor enhancement [8, 40].

The therapeutic effects of 2L involve comprehensive modulation of neuroendocrine systems and molecular pathways underlying stress resilience and neuroplasticity. The normalization of corticosterone levels following 2L treatment represents a critical therapeutic outcome, as HPA axis dysregulation is central to stress-related pathology [41]. The significant reduction in elevated corticosterone demonstrates that gut microbiota modulation can directly influence neuroendocrine function through vagal and humoral pathways [41, 42]. Beyond classical stress hormone regulation, 2L treatment influenced multiple neuromodulatory systems, as evidenced by the concurrent normalization of β-endorphin and oxytocin levels. The elevation of serum serotonin in the 2L group is particularly significant, given that serotonergic dysfunction is implicated in both depression and anxiety disorders [43]. While some gut bacteria can produce serotonin precursors, the observed increase may also reflect improved gut-brain communication and enhanced central serotonergic function [44]. This systemic serotonergic enhancement was further supported by the restoration of hippocampal serotonin receptors (HTR1A and 5HT7R), which play crucial roles in mood regulation, anxiety, and stress response [45].

The molecular mechanisms underlying 2L's neuroprotective effects involve restoration of key neuroplasticity markers and attenuation of stress-induced pathological cascades. The restoration of hippocampal BDNF expression represents a fundamental mechanism, as BDNF is essential for neuronal survival, synaptic plasticity, and stress resilience [46]. The ability of 2L to restore BDNF levels suggests that gut microbiota modulation can enhance neuroplasticity and promote neuronal recovery following stress-induced damage.

Furthermore, 2L treatment attenuated stress-activated kinase pathways (ERK1/2 and JNK1/2), which are activated by chronic stress and contribute to neuroinflammation, synaptic dysfunction, and neuronal damage [47, 48]. This anti-inflammatory mechanism, combined with the restoration of blood-brain barrier integrity through tight junction protein upregulation, provides comprehensive neuroprotection against stress-induced pathology.

The normalization of DNA methyltransferases (DNMTs) reveals an additional layer of therapeutic complexity, indicating that 2L treatment influences epigenetic regulation. Chronic stress can induce persistent epigenetic modifications that alter gene expression patterns and contribute to long-term behavioral and physiological changes [49]. The restoration of DNMT expression suggests that probiotic intervention may help reverse stress-induced epigenetic alterations and promote adaptive gene expression patterns, potentially providing long-lasting therapeutic benefits beyond the treatment period.

The genus-level microbiota analysis reveals specific bacterial alterations that may contribute to the therapeutic effects of 2L treatment. The successful colonization confirmed by increased *Limosilactobacillus* abundance demonstrates strain-specific establishment, which is essential for sustained therapeutic effects [50]. The trend toward increased *Butyricicoccus* abundance is particularly relevant, as this genus produces butyrate, a short-chain fatty acid with well-established anti-inflammatory and neuroprotective properties [51, 52]. The restoration of beneficial bacteria (*Faecalibaculum* and *Akkermansia*) while normalizing pathogenic taxa (*Prevotella*) suggests that 2L treatment promotes overall microbiota homeostasis rather than simply adding probiotic organisms. *Akkermansia* is particularly noteworthy for its role in maintaining intestinal barrier integrity and metabolic health [53], while *Faecalibaculum* has been associated with anti-inflammatory effects and protection against intestinal pathology [54].

The multifaceted effects of 2L demonstrated in this study suggest significant therapeutic potential for stress-related disorders. The ability to simultaneously target gut barrier function, local and systemic inflammation, HPA axis regulation, and neuroplasticity represents a comprehensive approach that addresses multiple pathophysiological mechanisms underlying these conditions [14, 18, 55].

While this study provides comprehensive evidence for the therapeutic effects of 2L, several limitations should be acknowledged. The UCMS model, while well-established, may not fully recapitulate the complexity of human stress-related disorders. The mechanisms of strain-specific effects require further elucidation, particularly regarding the production of bioactive metabolites and their direct effects on host physiology. Long-term safety studies and evaluation of therapeutic efficacy in other stress-related disorder models would strengthen the evidence base for clinical translation.

## Conclusion

This study demonstrates that *L. fermentum* 2L provides comprehensive protection against chronic stress-induced pathology through gut-brain axis modulation. The therapeutic effects involve restoration of gut microbiota homeostasis, strengthening of intestinal barrier function, normalization of HPA axis activity, and enhancement of hippocampal neuroplasticity. These findings support the therapeutic potential of targeted probiotic interventions for stress-related neuropsychiatric disorders and highlight the importance of gut-brain communication in mental health. The multifaceted mechanisms of action demonstrated here provide a strong foundation for clinical development of this probiotic strain as a novel therapeutic approach for stress-related disorders.

## Supplemental Materials

Supplementary data for this paper are available on-line only at http://jmb.or.kr.



## Figures and Tables

**Table 1:** Acid and bile tolerance and intestinal adhesion and antioxidant activities of selected *Lactobacillus* strains.