# Supplementary material for: Limosilactobacillus fermentum 2L Ameliorates Chronic Stress-Induced Neuroinflammation through Gut-Brain Axis Modulation in Mice
Source: J Microbiol Biotechnol. 2025 Nov 26;35:e2509035. doi: 10.4014/jmb.2509.09035 (PMC12685588; doi:10.4014/jmb.2509.09035)
Supplement: Supplementary file 1 [file jmb-35-e2509035-supple.pdf]

**J. Microbiol. Biotechnol. 2025. 35: e2509035**
https://doi.org/10.4014/jmb.2509.09035

# Supplementary Figure and Table

## *Limosilactobacillus fermentum* 2L ameliorates chronic stress-induced neuroinflammation through gut-brain axis modulation in mice

Jae Yeon Joung,[1,2+] Sejin Cheon,[1+] Jae Gwang Song,[3] Chaeeun Han,[3] Jeong Seok So,[3] Jong Kook Moon,[4] Hyung Wook Kim,[3*] and Sae Hun Kim[1,2*]

[1] College of Life Sciences and Biotechnology, Korea University, Seoul 02841, Republic of Korea

[2] Institute of Life Sciences and Natural Resources, Korea University, Seoul 02841, Republic of Korea

[3] Department of Integrative Bioscience and Biotechnology, Sejong University, Seoul 05006, Republic of Korea

[4] BTSynergy Co., Ltd., Cheongju, Republic of Korea

**[+]Jae Yeon Joung and Sejin Cheon contributed equally to this study.**

[*]**Corresponding author:** Sae Hun Kim and Hyung Wook Kim

**Table S1. Primer sequences used in this study.**

| Primer | Sequence | |
|---|---|---|
| GAPDH | Forward: | AGGTCGGTGTGAACGGATTTG |
| | Reverse: | TGTAGACCATGTAGTTGAGGTCA |
| IL-1β | Forward: | GCAACTGTTCCTGAACTCAACT |
| | Reverse: | ATCTTTTGGGGTCCGTCAACT |
| IL-6 | Forward: | TAGTCCTTCCTACCCCAATTTCC |
| | Reverse: | TTGGTCCTTAGCCACTCCTTC |
| IL-10 | Forward: | GCTCTTACTGACTGGCATGAG |
| | Reverse: | CGCAGCTCTAGGAGCATGTG |
| NF-κB | Forward: | CCAAAGCTCCCGAAACCAATC |
| | Reverse: | GAGTAGCCGCCGTAATAGGC |
| ZO-1 | Forward: | GCCGCTAAGAGCACAGCAA |
| | Reverse: | TCCCCACTCTGAAAATGAGGA |
| ZO-2 | Forward: | ATGGGAGCAGTACACCGTGA |
| | Reverse: | TGACCACCCTGTCATTTTCTTG |
| Ocldn | Forward: | TTGAAAGTCCACCTCCTTACAGA |
| | Reverse: | CCGGATAAAAAGAGTACGCTGG |
| Cldn4 | Forward: | GTCCTGGGAATCTCCTTGGC |
| | Reverse: | TCTGTGCCGTGACGATGTTG |
| Cldn5 | Forward: | CAGTTAAGGCACGGGTAGCA |
| | Reverse: | GGCACCGTCGGATCATAGAA |
| Cldn12 | Forward: | ACTGCTCTCCTGCTGTTCGT |
| | Reverse: | TGTCGATTTCAATGGCAGAG |
| CREB | Forward: | AGCAGCTCATGCAACATCATC |
| | Reverse: | AGTCCTTACAGGAAGACTGAACT |
| BDNF | Forward: | TCATACTTCGGTTGCATGAAGG |
| | Reverse: | AGACCTCTCGAACCTGCCC |
| NPY | Forward: | ATGCTAGGTAACAAGCGAATGG |
| | Reverse: | TGTCGCAGAGCGGAGTAGTAT |
| Nr3c1 | Forward: | AGCTCCCCCTGGTAGAGAC |
| | Reverse: | GGTGAAGACGCAGAAACCTTG |
| Dnmt1 | Forward: | AAGAATGGTGTTGTCTACCGAC |
| | Reverse: | CATCCAGGTTGCTCCCCTTG |
| Dnmt3a | Forward: | CTGTCAGTCTGTCAACCTCAC |
| | Reverse: | GTGGAAACCACCGAGAACAC |
| Dnmt3b | Forward: | AGCGGGTATGAGGAGTGCAT |
| | Reverse: | GGGAGCATCCTTCGTGTCTG |
| HTR1A | Forward: | GACAGGCGGCAACGATACT |
| | Reverse: | CCAAGGAGCCGATGAGATAGTT |
| HTR2A | Forward: | TAATGCAATTAGGTGACGACTCG |
| | Reverse: | GCAGGAGAGGTTGGTTCTGTTT |

**Table S2. Antibodies used in this study.**

| Antibody | Host | Supplier | Dilution |
|---|---|---|---|
| β-actin | Mouse | Invitrogen | 1:500 |
| BDNF | Rabbit | Bioss | 1:500 |
| 5HT7R/SR-7 | Rabbit | Invitrogen | 1:500 |
| Claudin5 | Mouse | Invitrogen | 1:500 |
| ERK1/2 | Mouse | Invitrogen | 1:500 |
| p-ERK1/2 | Rabbit | Invitrogen | 1:500 |
| JNK1/2 | Mouse | Invitrogen | 1:500 |
| p-JNK1/2 | Mouse | Invitrogen | 1:500 |
| PPARα | Mouse | Invitrogen | 1:500 |

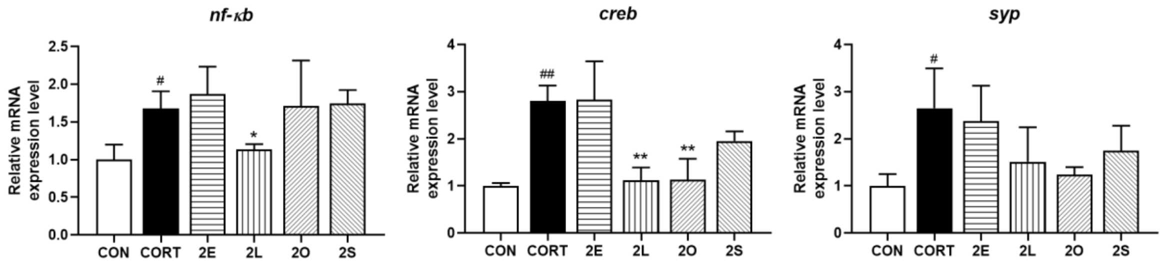


**Fig. S1. Effects of *L. fermentum* strains on inflammatory and neurological marker expression in corticosterone-treated SH-SY5Y cells.** qRT-PCR analysis of *nf-κB*, *creb*, and *syp* mRNA expression levels. Data are presented as mean ± SEM from three independent experiments. Statistical significance was determined by one-way ANOVA followed by Tukey's multiple comparison test. $^{\#\#}p < 0.005$ compared to CON group. $^{*}p < 0.05$, $^{**}p < 0.005$ compared to CORT-treated group. CON, untreated control; CORT, corticosterone-treated control; 2E, 2L, 2O, 2S, *L. fermentum* strains.
